# Determinants of Virus Variation, Evolution, and Host Adaptation

**DOI:** 10.3390/pathogens11091039

**Published:** 2022-09-13

**Authors:** Katherine LaTourrette, Hernan Garcia-Ruiz

**Affiliations:** 1 Nebraska Center for Virology, University of Nebraska-Lincoln, Lincoln, NE 68583, USA; 2 Department of Plant Pathology, University of Nebraska-Lincoln, Lincoln, NE 68583, USA; 3 Complex Biosystems Interdisciplinary Life Sciences Program, University of Nebraska-Lincoln, Lincoln, NE 68583, USA

**Keywords:** virus evolution, co-evolution, host adaptation, mutation, reassortment, recombination, selection pressure, quasispecies

## Abstract

Virus evolution is the change in the genetic structure of a viral population over time and results in the emergence of new viral variants, strains, and species with novel biological properties, including adaptation to new hosts. There are host, vector, environmental, and viral factors that contribute to virus evolution. To achieve or fine tune compatibility and successfully establish infection, viruses adapt to a particular host species or to a group of species. However, some viruses are better able to adapt to diverse hosts, vectors, and environments. Viruses generate genetic diversity through mutation, reassortment, and recombination. Plant viruses are exposed to genetic drift and selection pressures by host and vector factors, and random variants or those with a competitive advantage are fixed in the population and mediate the emergence of new viral strains or species with novel biological properties. This process creates a footprint in the virus genome evident as the preferential accumulation of substitutions, insertions, or deletions in areas of the genome that function as determinants of host adaptation. Here, with respect to plant viruses, we review the current understanding of the sources of variation, the effect of selection, and its role in virus evolution and host adaptation.

## 1. Introduction

As the global population approaches 9 billion people, current food production is threatened by climate change and decreasing land availability, making food security an increasing concern [1]. One approach to addressing these concerns is to reduce crop loss caused by plant pathogens, including viruses. Plant viruses affect economically important staple, cash, non-cash, and secondary-staple crops, resulting in over USD 30 billion in crop loss annually [2,3]. Further, plant viruses make up almost 50% of the emerging and reemerging infectious diseases [2,4]. There is a rise in virus outbreaks in part because climate change increases the geographic areas where viruses and vectors overlap [5,6,7]. This problem is exacerbated by viruses spreading into new areas through global trade and agricultural expansion [2,8].

Plant viruses are obligate parasites that use host processes and resources to replicate and spread. They consist of single- or double-stranded DNA or RNA contained within a virion. To enter the host, plant viruses need to bypass the cuticle and cell wall [9]. This can take place either through a virus vector feeding on the plant or through mechanical means. Successful infection only occurs when there is a compatible interaction between a host and a virus [6,10]. Insects are the most numerous vectors, with aphids, whiteflies, and leafhoppers being the most common [11]. However, mites, fungi, and nematodes are important vectors for some viruses [11]. Plant viruses can also be spread through grafting, seed, and vegetative cuttings [6]. Once inside the cell, viruses uncoat, and their genetic information, for positive strand RNA viruses is translated by the host machinery, leading to virus replication and virion formation [9]. In a continuous cycle, infection begins at a single-cell level, spreads cell to cell through the plasmodesmata, and eventually leads to long distance movement throughout the host [12].

Plants defend against viruses through multiple mechanisms, including gene silencing [13], autophagy [14], and Resistance (R) genes [15]. R genes interact with pathogen avirulence genes (avr) that activate host defenses, including programmed cell death [15]. Dominant and recessive R genes have been identified in wild relatives and introduced through plant breeding into commercial cultivars of important crops such as tomato, soybean, and potato [16,17,18]. However, new virus variants and species can quickly break host resistance, especially when virus resistance is dependent on a single gene [19,20]. This phenomenon occurs when in key areas of the virus genome, mutations accumulate that provide a selective advantage [19,20,21].

**Figure 1:**
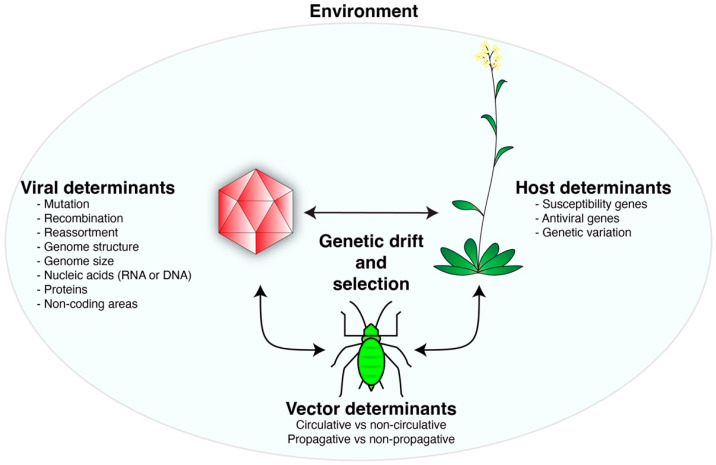
A model of virus evolution driven by the interactions between a virus, host, and vector. These interactions, combined with genetic drift and selective pressure, generate new variants. Contributing variables are indicated for viruses, vectors, and host plants.

The establishment of infection involves a compatible interaction between a virus and a host in a favorable environment (Figure 1). During replication, viruses may recombine, generate mutations, and, for RNA viruses, exist as a cloud of genetic variants (quasispecies) [22,23,24,25]. There is natural genetic diversity in host plants and vectors and variation in environmental conditions [20,26]. Accordingly, viruses are forced to interact with and adapt to genetically diverse host and vector factors in diverse environments. Thus, host, vector, and environmental factors, through genetic drift and selection, drive virus evolution (Figure 1). Virus evolution is established as changes in the genomic structure of a viral population, causing the emergence of new viral variants or species with novel biological properties. 

This review provides a broad view of plant virus evolution and host adaptation through the lenses of genetic variation and selection. It is focused on the contributions of mutation, reassortment, and recombination to the generation of genetic diversity and on selection constraints imposed by vectors, hosts, and environmental factors, as driving forces for the emergence of new variants and new viral species with novel biological properties. Contributions of mutation, reassortment, and recombination to genetic variation and the balancing effects on selection and bottlenecks on reducing genetic variation have been reviewed [27].

### 1.1. Plant Virus Genome Organization

**Figure 2:**
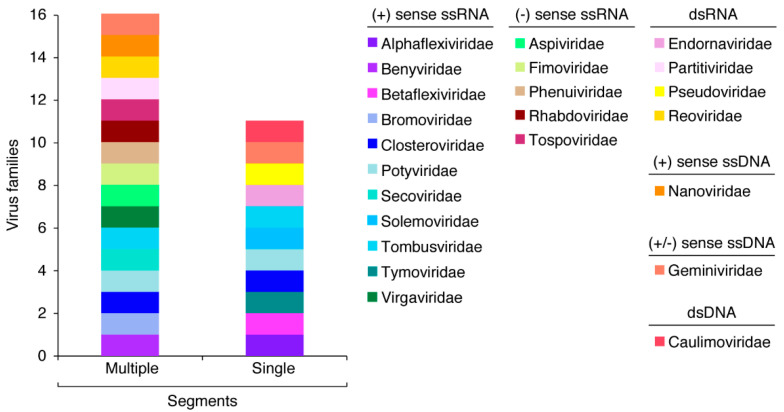
Frequency of plant virus families with multiple and single genomic segments. Some families contain species in both categories. Families are color coded.

Plant viruses are a diverse group of species with a broad array of genome organization and gene expression strategies classified into taxonomic families, each with several genera and species. Whether measured by family, genus, or species, the number of RNA viruses is greater than the number of DNA viruses, with most plant viruses being (+) sense ssRNA viruses [28]. Virions might be enveloped or not enveloped, with diverse morphologies, genome sizes, and organization [29]. These characteristics are most similar between viruses in the same taxonomic family or genus but do vary across families. The most common genome structure is a segmented genome (Figure 2). The families *Potyviridae, Closteroviridae, Tombusviridae*, and *Geminiviridae* contain both monopartite and bipartite species (Figure 2). Single-stranded genomes and segmented genomes have been linked to a wider host range [30]. Segmented genomes have shown to support higher mutation rates, particle stability, and genetic exchange through reassortment [31]. These genome structures are the most common across plant viruses, suggesting there is an evolutionary benefit.

### 1.2. Virus-Plant Co-Evolution

Plant viruses rely on host factors to replicate, move cell to cell, and travel systemically, but there is natural variation in those host factors [20,32]. Accordingly, plant viruses co-evolve with their host [33,34] and establish a balance between genomic functionality and the bottlenecks imposed by host genetic diversity, host compatibility, antiviral defense, and the fitness cost to the virus associated with host–range expansion or host specialization [10,30,35,36]. The coevolution of viruses with their hosts is tightly linked to an arm race with the host defense system and the availability of host factors for pro-viral functions in multilayered cooperation [35]. The arms race of coevolution includes multiple layers of defense and counter-defense in which the hosts constantly evolve new means of defense that viruses constantly evolve to evade or suppress [13]. In a clear indication of cooperation, viruses co-op host genes for counter-defense, replication, and movement, and host cells recruit viral genes for diverse roles [10,35].

Viruses evolve in a host-specific manner, accumulating mutations that can increase or decrease fitness, virulence, or infectivity in a certain host [36,37,38]. Interestingly, not all areas of the virus genome accumulate mutations at the same rate. Instead, mutations preferentially accumulate in parts of the genome that are determinants of host adaptation [20,39,40,41].

When a virus is spread to a plant, infection can only occur if the interaction is compatible. This can occur when a host lacks factors, such as R genes, that would otherwise prevent infection from occurring. For example, mutating the BRI1-associated kinase 1 (BAK1) results in increased virus susceptibility [42]. Compatibility can also be mediated by the availability of susceptibility host factors essential for virus replication and by the balance between antiviral defense and the suppression of the evasion of antiviral defense [13]. Mutations in host-susceptibility genes may break the interaction with a critical viral component, reducing or eliminating virus replication or movement [43,44]. In addition, hosts can accumulate mutations in genes that mediate antiviral defense. For example, the dominant-resistance gene Ry(o)*phu* in *Solanum tuberosum* groups *Phureja* and *Tuberosum* provides protection to potato virus Y (PVY) by blocking viral replication and movement [44]. Similarly, melon accession PI 164323 is resistant to cucumber vein yellowing virus. However, a single amino acid change in the VPg coding region restored compatibility and broke resistance [45]. 

Often, viruses have a host range limited to a single taxonomic plant family and are not able to infect across multiple plant families [30]. This is explained by fitness optimization to a specific host (niche-filling model) [46]. However, infection across families and even across kingdoms might occur [47], forcing viruses to face strong adaptive selection pressure to maximize their fit to a new niche [46]. When viruses are exposed to genetically uniform hosts, local adaptation occurs [48]. Viruses become specialized to certain hosts due to adaptive tradeoffs where increased fitness in one host leads to decreased fitness in other hosts due to either epistatic interactions or antagonistic pleiotropy [49,50,51]. When tobacco etch potyvirus was sequentially passaged from *Nicotiana tabacum* into pepper, the virus accumulated genetic changes that increased virulence and coat protein accumulation in pepper at the cost of lesser virulence and accumulation in *N. tabacum* [33]. When not well-adapted to a host, a virus is less able to hijack the host resources for replication and movement [52]. Bridge hosts may allow the virus to accumulate mutations that support host range expansion without adaptive tradeoffs [53]. Plum pox virus accumulated VPg mutations as it moved from an *N. clevelandii*-adapted isolate to the bridge-host *Arabidopsis thaliana*, which allowed partial adaptation to *Chenopodium foetidum* [53]. 

Host response can modulate infection, as illustrated by the proteomic comparison between cucumber cultivars resistant to or susceptible to cucumber mosaic virus (CMV). The results showed that the cultivars differentially express photosynthetic, development, stress, and defense-related proteins during infection [54].

Host plants evolve and mutate, forcing viruses to adapt to genetically heterogenous host populations. In plants, ARGONAUTE 2 (AGO2) is an important component of antiviral gene silencing [55]. In *A. thaliana*, AGO2 shows a high degree of polymorphism that correlates with changes in susceptibility to potato virus X (PVX) [56]. In ecotype Colombia, AGO2 confers resistance to PVX, imposing selection pressure on the virus. In contrast, in ecotype C24, AGO2 confers susceptibility to PVX, potentially imposing selection pressure on the host [56]. Although AGO2 diversity is not the result of co-evolution with viruses, this example illustrates diversity in a host gene, with antiviral activity imposing selection pressure on a virus. While viruses can evolve and adapt rapidly, their hosts may ultimately shape their longer-term evolution [46,57]. 

### 1.3. Virus-Vector Co-Evolution

Plant viruses are vectored from plant to plant by a wide variety of insects, nematodes, mites, and fungi [58]. Viruses can be transmitted in a circulative or non-circulative manner with non-persistent, semi-persistent, or persistent subcategories [59]. Virus and vector interactions are highly specialized and under strong selection pressure, in part, as result of co-evolution [11,60,61,62]. For geminiviruses, capsid phylogenies reflect the phylogeny of their vectors rather than that their host plant species, supporting the model that specificity of virus–vector interactions is more stringent than the specificity of virus–plant specificity [63].

Plant viruses often have a narrow vector range: ~60% are vectored by a single species [58]. Poor compatibility between vector and virus can result in the low incidence or elimination of a virus [59]. In southern California, lettuce infectious yellows virus incidence dropped significantly after biotype A of whitefly *Bemisia tabaci* was replaced by biotype B, a poor vector for the virus [64]. Additionally, mutations in viral proteins that interact with vector factors can eliminate or alter transmission, creating strong selection pressure [65,66]. For example, a single amino acid in the coat protein of squash leaf curl China virus is correlated with increased transmission by *Bemisia tabaci* Asia-1 compared with Asia-II-1 [67].

Plant viruses can directly or indirectly alter vector behavior and physiology by inducing transcriptional changes in metabolism-related genes in their vectors [68], which alters virus transmission [69]. Viruses transmitted in a nonpersistent manner indirectly mediated positive fitness effects on their vector through changes in the host plant [70]. Vector *Aphis gossypii* feeds more frequently on virus-infected plants (single or mixed infections), and both *Frankliniella occidentalis* and *A. gossypii* showed higher fitness after feeding on virus-infected plants [70,71]. Tomato yellow leaf curl virus (TYLCV) directly affects *Bemisia tabaci* settling, probing, and feeding behavior, leading to increased transmission efficiency and spread [72]. Tomato spotted wilt tospovirus infection alters host gene expression, resulting in higher total free amino acid content, making it nutritionally advantageous and better suited for thrips vector colonization [71]. Illustrating co-evolution, the specialist aphid *Lipaphis erysimi* has greater population growth in turnip mosaic virus (TuMV)-infected plants compared with noninfected plants. In contrast, the population growth of a generalist aphid vector *Myzus persicae* was similar in TuMV-infected and healthy hosts [73]. However, some viruses trick vectors into feeding on poor-quality hosts through volatile emissions [61]. 

### 1.4. The Environment and Virus Evolution

Environmental conditions directly impact virus infection, prevalence, evolution, and host interactions. These include abiotic factors like temperature, water stress, CO_2_ levels, ecology, community makeup, and population heterogeneity [5,26,74,75]. Viruses, hosts, and vectors exist within a given ecosystem, which can impact the spread, disease risk, and symptoms of virus infections. Accordingly, virus evolution is also impacted by ecological factors such as host abundance and species richness, which are affected by climate change and land use [74,76]. In some cases, a loss of biodiversity in host communities is correlated with increased disease [75]. However, adding genetic diversity can amplify disease incidence if hosts are susceptible or host density increases [77]. Additionally, human management and changes in land use result in a higher disease risk due to lowered biodiversity, reduced host diversity, and increased host density [74,78]. In coordination, some virus families are found most prevalently in cultivated areas [79]. 

While plant viruses are studied primarily in agricultural systems, they are highly prevalent in wild plant populations and often lead to asymptomatic infections, creating possible reservoirs that contribute to virus evolution [76]. Virus infection can be beneficial rather than harmful to hosts experiencing cold damage [80] or drought [81], and environmental conditions alone can shift viruses from being antagonistic to being mutualistic to the host [82]. The environment is likely to be increasingly important for plant virus infections as climate change continues to alter vector, host, and virus properties [5,7]. 

### 1.5. Variation, Bottlenecks, Genetic Drift, and Selection Pressure in Virus Populations

**Figure 3:**
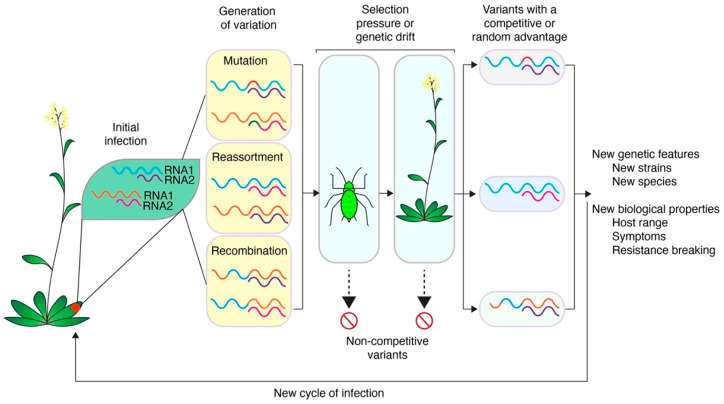
Generation of genetic variants through mutation, reassortment, and recombination. After initial infection, diverse genomes within the same host exist and may mutate, reassort, or recombine. These variants are exposed to genetic drift and selection pressure by host and vector factors. Random or competitive variants are passed on and may become fixed in the population and emerge as new strains or species with novel biological properties.

Viruses exist as a cloud of related sequences, called a quasispecies, rather than a single consensus [23,24,25]. This genetic variation is the basis for the evolution and adaptation of viruses to hosts, vectors, or their environment [83]. During normal replication, genetic variation is generated through mutation, reassortment, and recombination (Figure 3). These processes underlie the ability of plant viruses to generate and maintain genetic diversity, key to circumventing and adapting to host and vector processes [83]. Mutations can occur through substitutions, insertions, or deletions introduced during the replication by RNA-dependent RNA polymerases that lack proofreading activity [84]. Reassortment occurs between segmented viruses where entire genetic segments are exchanged between related viruses [85]. Recombination involves the transfer of genetic material between parental genomes, often by template switching during genome replication, which leads to new sequence combinations [86,87]. 

Host and vector factors select for or against variants produced through mutation, recombination, or reassortment [23,83]. Unfit variants are removed and variants with a competitive advantage are fixed in the population (Figure 3). Characteristics of an RNA virus population are determined by interactions between co-infecting viruses in mixed infections, variants in a quasispecies, and selection acting on individual variants, leading to population-level changes [24,25]. Illustrating this, quasispecies cloud sizes change during host shifts, and quasispecies diversity varies across related viruses [88,89]. Accordingly, quasispecies increase the probability a virus can adapt to new hosts, vectors, environments, and challenges during infection [24]. 

Genetic drift is the change in the frequency of variants in a virus quasispecies due to random chance [90]; some variants may disappear, while some initially rare variants may become more frequent or fixed in the population [90,91]. Similar to selection, genetic drift can decrease variation within a given population while simultaneously increasing the genetic distance between viral populations. Unlike selection, genetic drift results in variants moving onward in the population through random effects rather than a fitness bonus [90,91,92].

Genetic bottlenecks are evolutionary events that cause a sharp reduction in the size and genetic diversity of a virus population in a stochastic manner [90]. The fittest variants may fail to be transmitted due to the small population size generated by these bottleneck events [90]. In this way, bottleneck events counterbalance the genetic variation generated by mutation, reassortment, and recombination [90]. For viruses, vector transmission, the environment, and availability of susceptible hosts may impose genetic bottlenecks that decrease virus genetic diversity [93]. For example, horizontal transmission by aphids caused a significant bottleneck of an artificial population of CMV where variants were consistently lost during transmission [93]. Additionally, bottleneck events occur as a virus moves between hosts and systemically within a host [90,93,94]. These bottlenecks are often extremely narrow, resulting in a small number of variants establishing a new population and potentially generating new strains [88,90]. This founder effect is a form of genetic drift and results in a randomized group of virus sequences moving forward in the population. Founder effects have the potential to eliminate advantageous variants or propagate less advantageous variants [90]. Thus, selection and drift can have opposite effects on virus evolution [91,94]. However, founder effects produced by genetic bottlenecks can result in new species, making bottlenecks an important part of virus evolution [90]. Both processes are important to consider, but distinguishing the effects of the two processes is difficult. Regardless, the generation of variation and maintenance of a diverse quasispecies cloud provides a larger genetic pool for evolutionary forces to act upon irrespective of the fitness benefits or costs to any specific variant.

After selection or bottleneck events, variants become established in the population and emerge as new virus strains or species with novel biological properties, such as pathogenicity or host range expansion [24,35,89]. Therefore, the continuous emergence of new species and strains with novel properties is, in part, explained by natural variation generated through mutation, reassortment, and recombination.

### 1.6. Host Adaptation

Host adaptation is the development of higher fitness in a host, mediated by the accumulation of genetic changes in key parts of the viral genome. New species, strains, or genetic variants may be better suited to a particular host and thus be selected for in the population [21,45,48]. Over time, the virus will evolve towards an optimal balance with the host: replicating and moving without killing the host before transmission [63]. Similarly, on the host side, tolerance can occur when virus and host fitness are balanced [95]. When related viruses have differences in features such as host range, symptoms, or resistance, one approach to studying viral determinants of host adaptation is to identify the changes in the genome that mediate the contrasting differences. This approach first identifies the novel property and then identifies the genetic changes. For example, a narrow host range strain of CMV was identified, which suggests lack of variation in a viral determinant of the host range [96]. Pathogenicity and host range were mapped to the 2b protein by swapping the gene between different strains [96,97]. A consequence of this approach is that results are often placed within the context of a particular virus in a specific spatial or temporal environment [20]. 

## 2. Mutation

Mutations occur through nucleotide insertions, deletions, or substitutions in viral genomes. Mutations initially result in small-scale changes in the genome sequence. However, accumulations of mutations result in large-scale changes, generating novel genomes evident as new strains with novel properties. RNA viruses have a substitution rate of 10^−6^ to 10^−4^ substitutions per nucleotide per cell infection, compared with 10^−8^ to 10^−6^ substitutions per nucleotide per cell infection for DNA viruses [98]. That is, RNA viruses mutate at a rate that is 100-fold faster than DNA viruses. This is explained by the low fidelity and lack of proofreading activity of RNA-dependent RNA polymerases that replicate viral RNA genomes compared with DNA polymerases [84]. However, confounding factors such as the transmission method and cell tropism can alter substitution rates [99,100]. Single-stranded genomes and viruses with small genomes also show higher mutation rates, blurring the evolutionary advantages of RNA vs DNA viruses [98]. As a virus replicates, unique mutations accumulate in each of the progeny genomes, creating genomes distinct from the parent sequence [101]. Strong intracellular selection and competition occur as these unique sequences replicate and move within the host [101].

Correct virus orthology is identified by nucleotide or amino acid similarity cutoffs [102]. Within a species, mutations have been directly implicated in the generation of new biological properties. For example, a single amino acid mutation allows plum pox virus C and PVY to host jump [103,104]. A single mutation cannot result in a new species [105]. In contrast, a single mutation may result in a new strain if it changes the biological properties of the virus [91,106]. Specific mutations have also allowed viruses to break resistance in important crops such as soybean, melon, and pepper [19,107,108,109]. Further, mutations can alter symptoms in a virus-infected host [110,111], alter the function of virus factors [112], or impact vector transmission [65]. Biologically, mutations in the virus genome are necessary to accommodate the variation present in host proteins [56,113]. Indeed, the ability of a virus genome to tolerate mutations (mutational robustness) is considered an evolutionary advantage in enhancing the establishment of infection in genetically diverse hosts and vectors [36,83].

Experimental mutational analyses can identify the relationships between specific amino acids, protein function, and biological properties. Targeting these conserved amino acids or general areas can result in loss of function, gain of function, or altered symptomatology or virus orthography [110,111,112]. For example, a single amino acid mutation in papaya ringspot virus identified using mutational analyses alters host specificity [114]. Another approach to studying the role of mutation in virus evolution is to identify variation between strains that exhibit different characteristics [115]. However, a negative aspect of this approach is that it is unlikely to identify single mutations that can be traced to a specific biological role. 

## 3. Reassortment

Reassortment, formally called pseudorecombination, occurs when two related viruses co-infect a cell and entire RNA or DNA segments are swapped [116] (Figure 3). Reassortment only occurs in viruses with a segmented genome [91]. Within plant virus families, segmented genomes are the most common genome structure (Figure 2). Reassortment has been documented in almost every plant virus family with a segmented genome and has been responsible for the emergence of new strains with novel properties, including severe symptoms, pathotype differentiation, and resistance breaking (Table 1).

Mixed infections are more likely to occur when viruses and vector have overlapping temporal and spatial ecological niches [132,133]. Transmission mode, virus species, virus titer, host type, and original genetic variation of the virus population can impact the success of these mixed infections [134]. Reassortment can result in viruses containing novel combinations of segments with contrasting phylogenetic and evolutionary history and is especially important for generating genetic diversity in segmented (-) ssRNA viruses [116,135]. The fact that segmented viruses consistently evolve within and across families suggests that reassortment benefits viral fitness and evolution.

Segmented genomes may allow viruses to differentially express genes. During infection, virus segments gravitate towards the “setpoint genome formula” rather than being translated at an equal ratio [116]. This ratio suggests that segmented genomes in part evolved as a way to control the expression of viral proteins [136,137]. Further, this ratio changes from host to host. This indicates that host factors may also play a role in the regulation of the setpoint genome formula and that this process may be specifically advantageous when expanding into new hosts [136,137].

Variants produced through reassortment are subject to selection, and variants with a competitive advantage continue on in the population (Figure 3). Unfit variants are selected out through exposure to host, vector, and viral factors during cell-to-cell movement, systemic movement, and host colonization under bottleneck events [138,139]. Most reassortments result in less-fit variants due to the separation of co-adapted genes [140,141]. While viral genomic stability and the loss of reassorted variants is the status quo [142,143,144], variants that do survive in the population often are key for species differentiation and the evolution of novel characteristics (Table 1). 

Reassortment has led to the emergence of new viral strains with novel characteristics and biological properties and to the formation of specific clades in the population [85,145,146,147,148]. Reassortant viruses might be genetically diverse enough to break resistance [85,127], cause novel symptoms [149,150,151], alter host range [152,153], and evolve into new species. 

Examining reassortment in wild viral populations illustrates whether variants are emerging and remaining in the population. Measuring reassortment and recombination at the same time in a population allows for the comparison of their frequencies with fewer confounding factors (Table 2). Reassortment occurs at higher frequency than recombination for *Alfalfa mosaic virus*, in *Bunaviridae*, and in *Secoviridae* (Table 2). Reassortment frequency ranges from 3.33% to 55.17%, showing wide diversity across families, species, and likely specific wild populations (Table 2). 

## 4. Recombination

Recombination is a critical source of genetic variation, contributing to the continual evolution of viruses and to the emergence of new species [22]. It occurs both in DNA and RNA viruses [22,160]. In RNA viruses, recombination occurs when the RNA polymerase switches from an initial template (the donor) to a second template (the acceptor) during RNA synthesis, resulting in the fusion of noncontiguous sections of RNAs and creating new combinations of RNA sequences [22,116]. Recombination occurs in RNA replication compartments when both a donor and an acceptor RNA are recruited into the same replication compartment [87]. Template switching occurs in all RNA virus types but is less prevalent in double-stranded and negative-stranded RNA viruses [161]. Theoretically, through recombination, and possibly involving viral or cellular RNA-dependent RNA polymerases, viruses exchange genes with their hosts, and viral genes are often recruited for cellular functions [35,162].

Recombination does not occur randomly across the genome. Instead, recombination preferentially occurs at ‘hotspots’ within a virus genome [148]. It is possible that those hotspots represent areas of the genome that are mutationally robust and have no deleterious effects on the fitness of the novel progeny [83,163]. Most recombinants are less fit and will be selected out of the population (Figure 3) [163].

Across plant virus families, recombination has been identified except in *Endornaviridae, Partitiviridae, Pseudoviridae,* and *Aspiviridae* (Table 3). This is likely due to a low recombination rate in these virus families combined with a scarcity of research on the subject with these viruses. However, for the other plant virus families, recombination is a significant evolutionary force that shapes viral species and their features (Table 3). Unlike mutation and reassortment, one recombination event can create variants that are widely divergent from their parental sequences [164,165,166]. This can affect the evolutionary history of viruses by generating new species or strains, thereby shaking up population genetics (Table 3). Consistently, recombination results in viruses epidemiologically important because the recombinants are new species, are resistance breaking, or impact the long-term evolutionary history of the virus [22]. 

Several new species have been identified that are recombinants of either related or phylogenetically distant viruses. In the genus *Begomovirus*, tomato yellow leaf curl sardinia virus, tomato yellow leaf curl Axarquia virus, sweet potato leaf curl Canary virus, tomato leaf curl Mahé virus, and tomato leaf curl Yunnan virus are all new species created through recombination [106,164,182]. 

Recombination frequently shapes the trajectory of a virus population over time [173,179,184]. In Sudan, chickpea chlorotic dwarf virus showed extensive intra- and inter-strain recombination that, in combination with later diversification, led to the foundation of several strains [184]. Several diseases and epidemics have also been tied to recombinants [185,186,187]. This is likely connected with the fact that recombinant viruses can be resistance breaking [171,188]. For example, the spread and origin of Cassava mosaic disease in East Africa has been tied to a recombination event between two cassava mosaic geminiviruses, resulting in increased symptom severity [189]. Recombination can also allow a virus to infect a new host or to increase infectibility [105,165]. A recombination event between lettuce chlorosis virus and bean yellow disorder virus resulted in the recombinant lettuce chlorosis virus-SP that exhibited a shift in host range from lettuce, its original host, to green beans, a new host [185]. However, some recombination events result in decreased host range or infectibility, such as losing systemic infection [190]. This is consistent with the findings that recombination frequency is host specific [191]. Along with new host expansion, recombination can also change the symptoms or virulence of the virus [192,193]. These characteristics showcase the impact of recombination events that, while rare, can severely impact crop safety. 

Recombination in wild populations can result in the emergence of novel virus epidemics [86]. In studies measuring both recombination and reassortment, recombination frequency was greater for CMV, *Geminiviridae*, and *Nanoviridae* (Table 2). Recombination frequency ranged from 1.67% for AMV to 72.22% for faba bean necrotic yellows virus (FBNYV). For FBNYV, an ancestral recombination event led to all isolates from Spain and Tunisia being recombinants [157]. These observations show that viral population structure can be altered by a single recombination event. 

## 5. Genetically Stationary and Transient Viruses

A virus may move across the continuum between two conceptual genetically evolutionary stages, i.e. stationary (near equilibrium) and transient (far from equilibrium), changing its status as it is exposed to genetically diverse or identical hosts and environments. Viruses in a transient dynamic would be characterized by rapid genetic and phenotypic changes driven by directional or diversifying selection as they adapt to new hosts, vectors or environments. Naturally, these viruses are able to evolve and adapt better and faster to new hosts, vectors, and environments [194]. In general, both potyviruses and orthotospoviruses are constantly changing, and new variants or species are continually detected [42,43,83]. In contrast, viruses in stationary dynamics are characterized by viral populations near an optimal fitness in relation to their current hosts, vectors, and environments and therefore under strong purifying selection. The model is that stable viruses have a narrow host range, and they evolve and adapt slowly to new hosts, vectors, and environments [194]. Stationary viruses, such as maize chlorotic mottle virus, are specialized to infect a narrow range of hosts and vectors [195] and are likely to remain in the ecosystem as infection may benefit or not severely impact both the plant and the virus. Intrinsic properties related to virus genome structure [30], transmission [30], and quasispecies diversity [89] have been correlated with host range. Similarly, this model suggests that genetically transient viruses are mutationally robust and their populations are more likely to contain a variant able to adapt to a variable set of possible hosts without a fitness cost. In contrast, genetically stationary viruses have low mutational robustness and quasispecies diversity, making them less likely to contain advantageous variants [23,36]. While the links between genetic variability, host range, and mutational robustness are currently uncertain, the ability of a virus to adapt to diverse hosts and new environments and to gain novel biological features is likely connected to this evolutionary continuum.

## 6. Discussion

Viruses evolve through a combination of genetic variation in the population, genetic drift, and selection mediated by host, vector, and environmental factors (Figure 1). Because they replicate faster than their host can reproduce, viruses will continue to change, escaping host immune responses while maintaining functionality in different hosts and vectors [10,15]. Virus variants are produced naturally during the infection cycle through mutation, reassortment, or recombination, showcasing the continuous adaptation of viruses [196]. Based on their fitness or through founder events, variants are selected out of or fixed into viral genomes and virus population structure (Figure 3) [19,41]. Through this process, resistance-breaking virus strains or species with new host ranges or presenting novel symptoms can emerge and lead to epidemics threatening global food security [127,187]. Tomato brown rugose fruit virus (ToBRFV) emerged in 2015 after a recombination event between the major parent, tobacco mosaic virus strain Ohio, and the minor parent, tomato mild mottle virus [86]. Six amino acid substitutions in the movement protein of ToBRFV allowed it to overcome the Tm-2^2^ resistance gene, threatening global tomato production [197,198]. 

Virus taxonomy and species identification are important for virologists and the framing of their research [28,199]. High-throughput sequencing has revolutionized the identification of novel species in a wide range of hosts [74]. The known virome has subsequently expanded, and the need for classification has grown in coordination [200,201,202]. Currently, virus species are differentiated by sequence similarity cutoffs between related viruses [102]. However, reassortment and recombination often result in new virus species or strains with proteins exhibiting distinct evolutionary histories (Table 1 and Table 3) [105]. Reassortment and recombination are fundamental contributors to the evolution and differentiation of viruses and have high importance for the classification of new species and genera. When identifying species and strains, the genetic variation between and within species, the frequency of reassortment and recombination, and the phylogenetic history of viral proteins should be taken into account [102,105]. The identification of new viruses can take place in coordination with mutation, reassortment, and recombination analyses to identify the evolutionary history of the sequence and provide possible explanations for novel biological properties. Moving forward, more attention should be paid to investigating the sources of variation rather than simply showing it is present.

Measuring reassortment and recombination in wild populations remains difficult. However, occasional variants in wild populations emerge with significant economic and evolutionary effects (Table 1 and Table 3) [127]. Additionally, reassortment and recombination are commonly seen in phylogenetic and evolutionary studies of virus species over time [85,184] that show that these processes are occurring and shaping the genetic structures of wild virus populations. Further, wild population studies are more likely to reflect true recombination and reassortment frequencies because they incorporate all host, vector, and viral interactions. To support this, studies often see very different frequency rates between wild and experimental populations [203]. The frequency at which these events occur, while statistically rare [142], still results in epidemiologically important viruses and thus cannot be ignored. By examining wild population studies that measure both reassortment and recombination in the same environment, we can better understand the importance and frequency of these processes (Table 2). Further, wild and asymptomatic populations can further expand our knowledge of how these processes occur and the lasting impacts of these variants [24]. While reassortment and recombination show clear variation across families, between DNA and RNA viruses, and within families, they are still occurring within the natural population and thus should not be discredited as major evolutionary processes. 

## Figures and Tables

**Table 1:** Representative effects of reassortment organized by plant virus family.

*Family*	*Genus*	*Species*	Reassortment Effect	Reference
*Benyviridae*	*Benyvirus*	*Beet necrotic yellow vein virus*	Impact phylogenetic history	[117]
*Bromoviridae*	*Bromovirus, Cucumovirus, Ilarvirus, and Oleavirus*	18 species	Impact phylogenetic history	[118]
*Closteroviridae*	*Crinivirus*	*Tomato chlorosis virus*	Impact phylogenetic history	[119]
*Potyviridae*	*Bymovirus*	*Wheat yellow mosaic virus*	Pathotype differentiation	[120]
*Secoviridae*	*Comovirus*	*Bean pod mottle virus*	Severe symptoms	[121]
*Virgaviridae*	*Pomovirus*	*Potato mop-top virus*	Impact phylogenetic history	[122]
*Aspiviridae*	*Ophiovirus*	Blueberry mosaic associated ophiovirus*	Impact phylogenetic history	[123]
*Fimoviridae*	*Emaravirus*	Pigeonpea sterility mosaic emaravirus 1* and Pigeonpea sterility mosaic emaravirus 2*	Impact phylogenetic history	[124]
*Phenuiviridae*	*Tenuivirus*	*Maize stripe virus*	Impact phylogenetic history	[125]
*Rhabdoviridae*	*Dichorhavirus*	Orchid fleck dichorhavirus*	New strain	[126]
*Tospoviridae*	*Orthotospovirus*	Tomato spotted wilt orthotospovirus*	Resistance-breaking	[127]
*Partitiviridae*	*Alphapartitivirus, Betapartitivirus, Cryspovirus Deltapartitivirus, and Gammapartitivirus*	12 species	Impact phylogenetic history	[128]
*Reoviridae*	*Fijivirus*	*Rice black-streaked dwarf virus*	Impact phylogenetic history	[129]
*Nanoviridae*	*Babuvirus*	*Cardamom bushy dwarf virus*	Impact phylogenetic history	[130]
*Geminiviridae*	*Begomovirus*	*Tomato yellow leaf curl Mali virus and Cotton leaf curl Gezira betasatellite*	Severe symptoms	[131]

* Name is changed to reflect current ICTV taxonomy.

**Table 2:** Reassortment and recombination frequencies in wild viral populations. Frequencies of reassortment and recombination were calculated from papers studying both processes simultaneously in wild virus populations. Frequency was determined by the number of reassorted or recombined sequences identified divided by the total number of virus variants in the sampled population.

Family	Species	Reassortment Frequency	Recombination Frequency	Reference
*Bromoviridae*	*Alfalfa mosaic virus*	2/60 = 3.33%	1/60 = 1.67%	[154]
	*Cucumber mosaic virus*	4%	7%	[142]
*Tospoviridae*	Tomato spotted wilt orthotospovirus *	3/13 = 23.08%	2/13 = 15.38%	[155]
	Impatiens necrotic spot orthotospovirus *	6/18 = 33.33%	0/18 = 0%	[145]
*Geminiviridae*	*Pepper golden mosaic virus*	20/47 = 42.55%	27/47 = 57.45%	[156]
	*Pepper huasteco yellow vein virus*	11/42 = 26.19%	13/42= 31.00%	
*Nanoviridae*	*Faba bean necrotic yellows virus*	6/18 = 33.33%	13/18 = 72.22%	[157]
*Secoviridae*	*Broad bean wilt virus 1*	6/37 = 16.22%	2/37 = 5.41%	[158]
	*Broad bean wilt virus 2*	16/29 = 55.17%	2/29 = 6.70%	[159]

* Name is changed to reflect current ICTV taxonomy.

**Table 3:** Representative effects of recombination organized by plant virus family.

*Family*	*Genus*	*Species*	Recombination Effect	Reference
*Alphaflexiviridae*	*Potexvirus*	*Pepino mosaic virus*	Impact phylogenetic history	[167]
*Benyviridae*	*Benyvirus*	*Rice stripe necrosis virus*	Impact phylogenetic history	[168]
*Betaflexiviridae*	*Citrivirus*	*Citrus leaf blotch virus-Rec*	New species	[169]
*Bromoviridae*	*Cucumovirus*	*Cucumber mosaic virus*	Impact phylogenetic history	[170]
*Closteroviridae*	*Closterovirus*	*Citrus tristeza virus-RB*	Resistance-breaking	[171]
*Potyviridae*	*Potyvirus*	*Sudan watermelon mosaic virus*	New species	[172]
*Secoviridae*	*Nepovirus*	*Grapevine fanleaf virus*	Impact phylogenetic history	[173]
*Solemoviridae*	*Polerovirus*	*Sugarcane yellow leaf virus*	New species	[174]
*Tombusviridae*	*Tombusvirus*	*Pelargonium necrotic spot virus*	New species	[166]
*Tymoviridae*	*Tymovirus*	*Dulcamara mottle virus*	New species	[175]
*Virgaviridae*	*Tobamovirus*	*Ribgrass mosaic virus* *strain FSHS*	New strain	[176]
*Aspiviridae*	*NA*	*NA*	*NA*	*NA*
*Fimoviridae*	*Emaravirus*	*Pigeonpea sterility mosaic emaravirus 1 and Pigeonpea sterility mosaic emaravirus 2*	Impact phylogenetic history	[124]
*Phenuiviridae*	*Tenuivirus*	Rice stripe tenuivirus *	Impact phylogenetic history	[177]
*Rhabdoviridae*	*Alphanucleorhabdovirus*	*Eggplant mottled dwarf alphanucleorhabdovirus*	Impact phylogenetic history	[178]
*Tospoviridae*	*Orthotospovirus*	Iris yellow spot orthotospovirus *	Genotype separation	[179]
*Endornaviridae*	*NA*	*NA*	*NA*	*NA*
*Partitiviridae*	*NA*	*NA*	*NA*	*NA*
*Pseudoviridae*	*NA*	*NA*	*NA*	*NA*
*Reoviridae*	*Fijivirus*	*Southern rice black-streaked dwarf virus*	Impact phylogenetic history	[180]
*Nanoviridae*	*Babuvirus*	*Banana bunchy top virus*	Impact phylogenetic history	[181]
*Geminiviridae*	*Begomovirus*	*Tomato leaf curl Mahé virus*	New species	[182]
*Caulimoviridae*	*Caulimovirus*	*Cauliflower mosaic virus*	Impact phylogenetic history	[183]

* Name is changed to reflect current ICTV taxonomy.

## Data Availability

Not applicable.
